# Added Value of 3D Proton-Density Weighted Images in Diagnosis of Intracranial Arterial Dissection

**DOI:** 10.1371/journal.pone.0166929

**Published:** 2016-11-23

**Authors:** Jin Woo Kim, Na-Young Shin, Young Dae Kim, Seung-Koo Lee, Soo Mee Lim, Se Won Oh

**Affiliations:** 1 Department of Radiology, Inje University Ilsan Paik Hospital, Goyang, Gyeonggi, Korea; 2 Department of Radiology, Ewha Womans University School of Medicine, Seoul, Korea; 3 Department of Neurology, Yonsei University College of Medicine, Seoul, Korea; 4 Department of Radiology, Yonsei University College of Medicine, Seoul, Korea; 5 Department of Radiology, Soonchunhyang University Cheonan Hospital, Cheonan, Chungnam, Korea; Charite Universitatsmedizin Berlin, GERMANY

## Abstract

**Background:**

An early and reliable diagnosis of intracranial arterial dissection is important to reduce the risk of neurological complication. The purpose of this study was to assess the clinical usefulness of three-dimensional high-resolution MRI (3D-HR-MRI) including pre- and post-contrast T1-weighted volumetric isotropic turbo spin echo acquisition with improved motion-sensitized driven equilibrium preparation (3D-iMSDE-T1) and proton-density weighted image (3D-PD) in detecting dissection and to evaluate the added value of 3D-PD in diagnosing intracranial arterial dissection.

**Methods:**

We retrospectively recruited patients who underwent 3D-HR-MRI with clinical suspicion of arterial dissection. Among them, we selected patients who were diagnosed with definite dissection according to the Spontaneous Cervicocephalic Arterial Dissections Study criteria. For each patient, the presence of intimal flap, intramural hematoma, and vessel dilatation were evaluated independently by two neuroradiologists on each sequence. Interobserver agreement was assessed.

**Results:**

Seventeen patients (mean age: 41 ± 10 [SD] years; 13 men) were diagnosed with definite dissection. The intimal flaps were more frequently detected on 3D-PD (88.2%, 15/17) than on 3D-iMSDE-T1 (29.4%, 5/17), and post-contrast 3D-iMSDE-T1 (35.3%, 6/17; *P* = 0.006 and *P* = 0.004, respectively). No significant difference was found in the detection rate of intramural hematomas (59–71%) and vascular dilatations (47%) on each sequence. Interobserver agreement for detection of dissection findings showed almost perfect agreement (*k* = 0.84–1.00), except for detection of intimal flaps on pre-contrast 3D-iMSDE-T1 (*k* = 0.62). After addition of 3D-PD to pre- and post-contrast 3D-iMSDE-T1, more patients were diagnosed with definite dissection with the initial MRI (88.2% vs. 47.1%; *P* = 0.039).

**Conclusions:**

The intimal flap might be better visualized on the 3D-PD sequence than the 3D-iMSDE-T1 sequences, allowing diagnosis of definite dissection without follow-up imaging.

## Introduction

Cerebrovascular dissection can accompany various neurological events, including ischemic stroke or subarachnoid hemorrhage besides causing local symptoms such as headaches. Over 80% of these neurological complications occur in the first two weeks after onset of the first local sign or a transient ischemic attack [1, 2]. Therefore, an early and reliable diagnosis is important because early anticoagulation or antithrombotic treatment helps reduce the risk of neurological events [3].

For a decade, due to the introduction of black-blood (BB) techniques, direct wall delineation and identification or characterization of wall pathology has been possible in extracranial and intracranial arteries using noninvasive MRI [4–9]. Moreover, three-dimensional (3D) acquisition with submillimeter isotropic resolution enables better visualization of inherently curved and small intracranial arteries which in turn allows retrospective selection of the best imaging plane to detect wall pathology and minimizes the through-plane partial volume effect [10–12].

A few recent studies have suggested the clinical usefulness of 3D high-resolution MRI (3D-HR-MRI) in the diagnosis of intracranial dissection [12–14]. Although they consistently reported a high detection rate of intramural hematoma (IMH), opposite results were demonstrated for the intimal flap which is a pathognomic direct sign of intracranial dissection and one of the major criteria for definite dissection according to the Spontaneous Cervicocephalic Arterial Dissections Study (SCADS) criteria (Table 1) [15]. These studies all used 3D T1-weighted images with the motion-sensitized driven equilibrium preparation (MSDE) technique. This technique can effectively suppress blood flow which may help differentiate flow artifacts from intimal flaps [16, 17]. However, it can cause substantial signal loss, even with the recently proposed improved MSDE (iMSDE) technique which was designed to improve the signal-to-noise ratio (SNR), making it difficult to detect thin intimal flaps. Although the proton-density (PD) weighted sequence with a high SNR may detect intimal flaps missed on the iMSDE sequence, there has been no study conducted to prove its capability in detecting intracranial dissection [18]. Therefore, the aim of our study was to evaluate the added value of 3D-PD in detecting intimal flaps and diagnosing definite dissection without follow-up imaging or other imaging modalities (e.g., digital subtraction angiography [DSA] or computed tomography angiography [CTA]) in patients with intracranial spontaneous dissection.

**Table 1:** Diagnostic Criteria for Cervicocephalic Arterial Dissection.

Major criteria	“Double lumen” or “intimal flap” demonstrated on either DSA, MRI, MRA, CTA, or DUS “Pearl and string sign” or “string sign” demonstrated on DSA Pathological confirmation of arterial dissection
Minor criteria	“Pearl sign” or “tapered occlusion” demonstrated on DSA
	“Pearl and string sign”, “string sign” or “tapered occlusion” demonstrated on MRA
	“Hyperintense intramural signal” (corresponding to intramural hematoma) demonstrated on T1-weighted MRI
Additional criteria	Change in arterial shape demonstrated on either DSA, MRI, MRA, CTA, or DUS
	No other causes of arterial abnormalities
Definite dissection	Presence of one or more major criteria, or presence of one or more minor criteria and both of the additional criteria
Probable dissection	Presence of one or more minor criteria

## Materials and Methods

### Patients

We retrospectively recruited consecutive patients who underwent 3D-HR-MRI at our institution between April 2014 and December 2014 for clinically suspected arterial dissection. Clinical symptoms and signs which were considered to be related to arterial dissection were headaches, ischemic symptoms, or suspicious arterial dissection on previous conventional imaging studies such as 3D time-of-flight MR angiography (TOF-MRA) or CTA. Conventional imaging studies were performed either before or on the same day as 3D-HR-MRI and some patients underwent follow-up studies. Two board-certified neuroradiologists (N.Y.S. and J.W.K.) reviewed available baseline and follow-up (if applicable) conventional imaging studies and intracranial arterial dissection was diagnosed according to the SCADS criteria [15] (Table 1). Patients who met the criteria of definite dissection were selected for this study. In order to specifically select cases of spontaneous intracranial arterial dissection, patients with a history of related trauma, underlying disease associated with atherosclerosis, other vascular disease such as Moyamoya disease, and extra-cranial dissection were excluded. Disease stage at the time of 3D-HR-MRI was defined by the time passed since the onset of symptoms and was classified as acute (0–3 days), subacute (4–60 days), and chronic (>60 days). This retrospective study was approved by the Institutional Review Board of Severance Hospital and the Clinical Research Ethics Committee waived the requirement for written informed consent from the participants.

### Image Acquisition

The 3D-HR-MRI was performed using a 3.0-teslar system (Achieva, Philips Healthcare, Best, Netherlands) with a 32-channel SENSE head coil. In our 3D-HR-MRI protocol for arterial dissection, TOF-MRA was first performed to define the dissected arteries. Then, 3D-PD and pre- and post-contrast 3D T1-weighted volumetric isotropic turbo spin echo acquisition with iMSDE preparation (3D-iMSDE-T1) were acquired in a sagittal plane. Scan parameters for each sequence were as follows: (1) 3D-PD (repetition time [TR] / echo time [TE], 2000 ms / 32 ms; field of view [FOV], 240 x 240 x 200 mm; acquired matrix, 480 x 480 x 200; acquired resolution, 0.5 x 0.5 x 1.0 mm; reconstructed resolution, 0.5 x 0.5 x 0.5 mm; flip angle, 90°; turbo spin-echo factor, 80; refocusing angle, 120°; echo spacing, 6.5 ms; sensitivity encoding factor, 2.5 (phase) and 1 (section); number of signals acquired [NSA], 1; scan time, 12 minutes 58 seconds); (2) 3D-iMSDE-T1 (TR/TE, 500 ms / 31ms; FOV, 240 x 240 x 200 mm; matrix 480 x 480 x 400; acquired resolution, 0.5 x 0.5 x 0.5 mm; flip angle, 90°; turbo spin-echo factor, 16; refocusing angle, 50°; sensitivity encoding factor, 2 (phase) and 1.5 (section); NSA, 1; flow VENC, 1cm/s; all three directions of motion-sensitized gradients; scan time, 12 minutes 22 seconds); (3) Post-contrast 3D-iMSDE-T1 was acquired after intravenous injection of Gadoterate Meglumine (Dotarem^®^; Guerbet, Roissy CdG Cedex, France) at a dose of 0.1 mmol/kg using the same parameters as those for pre-contrast 3D-iMSDE-T1.

DSA images were acquired by using an Allura Xper FD20/20 (Philips Healthcare, Best, Netherlands). DSA images included not only conventional images (anteroposterior and lateral projected 2D images) but also 3D rotational images or VasoCT which is an iodine contrast-enhanced Cone-Beam CT [19].

### Image Analysis

Two board-certified neuroradiologists (N.Y.S. and J.W.K.) reviewed the 3D-HR-MRI images. For each sequence, we recorded the four major image findings of arterial dissection: (1) intimal flap or double lumen; (2) IMH; (3) vascular dilatation; and (4) wall enhancement. An intimal flap was defined as a linear structure crossing the arterial lumen located between the true lumen and false lumen. To distinguish the intimal flap from inflow artifacts which are usually located at the center of a vessel, we regarded the intimal flap as continuing linear structure to the arterial sidewall. IMH was defined as a crescent-shaped area of diverse signal intensity according to dissecting age. Vascular dilatation was defined as enlargement of the outer diameter of the affected vascular segment. Arterial wall enhancement was identified on the contrast-enhanced image, using the contralateral normal-appearing arteries as a reference standard. Disagreement between the two reviewers was resolved by consensus. Additionally, two observers recorded which sequence was preferred for detection of intimal flap, IMH, and vascular dilatation.

To assess the ability of 3D-PD to distinguish intimal flaps from flow artifacts, we also assessed the frequency of intimal flaps in the contralateral non-dissection side of all patients except for one patient with BA dissection.

### Statistical analysis

McNemar’s test was used for analyzing differences in the detection rate of the dissection findings among the 3D-PD and pre- and post-contrast 3D-iMSDE-T1 sequences. Interobserver agreement for detection of dissection findings was estimated using Cohen’s kappa coefficient. The results were interpreted according to the standards for strength of agreement proposed by Landis and Koch [20], as follows: poor agreement, ≤0; slight agreement, 0.1–0.20; fair agreement, 0.21–0.40; moderate agreement, 0.41–0.60; substantial agreement, 0.61–0.80; and almost perfect agreement, 0.81–1.0. Statistical analysis was performed using SPSS version 20.0 (SPSS, Inc., Chicago, IL, USA) and two-tailed *P*<0.05 was considered significant.

## Results

### Demographic and Clinical Characteristics

Thirty-eight patients underwent 3D-HR-MRI for evaluation of clinically suspected intracranial dissection. Twenty-one patients were excluded because they did not satisfy the diagnostic criteria for definite dissection. Eight patients were diagnosed with atherosclerosis, seven patients with extracranial dissection, and two patients with probable dissection according to the diagnostic criteria, while four patients showed no pathologic findings. A total of 17 patients (mean ± standard deviation [SD] age: 41 ± 10 years; 13 men and 4 women) were diagnosed with definite dissection: intimal flap or pearl and string sign on DSA (n = 9); intimal flap on CTA (n = 2); and change in arterial shape on follow-up imaging studies (n = 6). The arterial dissections were located in the posterior circulation in 12 patients and in the anterior circulation in 5 patients. Disease stages at the time of 3D-HR-MRI were acute (n = 1), subacute (n = 12), and chronic (n = 4). All 17 patients had unruptured intracranial arterial dissections. The clinical characteristics of the patients are summarized in Table 2.

**Table 2:** Characteristics of patients with intracranial artery dissection.

	All patients (n = 17)
Age (years) *	41 ± 10
Male	13 (76%)
Symptom	
Headache	10 (59%)
Dizziness	8 (47%)
Nausea/vomiting	6 (35%)
Motor weakness	3 (18%)
Sensory change	2 (12%)
Cerebellar sign	2 (12%)
Dissection stage	
Acute	1 (6%)
Subacute	12 (71%)
Chronic	4 (23%)
Dissection site	
Anterior circulation (distal ICA or MCA)	5 (29%)
Vertebral artery	11 (65%)
Basilar artery	1 (6%)

Unless otherwise indicated, data are numbers of patients. Numbers in parentheses are percentages within all patients.

* Values are means and standard deviations. ICA, Internal carotid artery; MCA, Middle cerebral artery.

### Detection of dissection findings on 3D-HR-MRI

The frequency of detection of dissection findings for each sequence on 3D-HR-MRI is summarized in Table 3. Intimal flaps were the most frequently detected on 3D-PD (88.2%, 15/17) with the highest interobserver agreement (*k* = 0.88) among the sequences. Moreover, 3D-PD was the most preferred sequence for identifying the intimal flap. The intimal flap was identified on the pre- and post-contrast 3D-iMSDE-T1 sequence in 29% (5/17) and 35% (6/17), respectively. Although IMH was more often detected on 3D-PD (70.5%, 12/17) than 3D-iMSDE-T1 (64.7%, 11/17) and post-contrast 3D-iMSDE-T1 (58.8%, 10/17), the difference did not reach statistical significance, and 3D-iMSDE-T1 was the most preferred sequence for identifying IMH. Vascular dilatations showed the same detection rate (47.1%, 8/17) on all sequences, and 3D-PD was the most preferred sequence for identifying vascular dilatation. On post-contrast 3D-iMSDE-T1, all dissected arteries revealed vascular enhancement regardless of the dissection stage.

**Table 3:** Frequency of dissection findings on 3D-HR-MRI.

Image findings of dissection		PD	iMSDE-T1	iMSDE-CE-T1	*P*1	*P*2	*P*3
**Intimal flap**		15 (88.2%)	5 (29.4%)	6 (35.3%)	**0.006**	**0.004**	1.0
	Interobserver agreement *Kappa*	0.88	0.62	0.84			
	Preferred sequence (R1/R2)	14/14	0/0	1/1			
**Intramural hematoma**		12 (70.5%)	11 (64.7%)	10 (58.8%)	0.5	1.0	0.5
	Interobserver agreement *Kappa*	1	1	0.88			
	Preferred sequence (R1/R2)	1/1	10/11	1/0			
**Vascular dilatation**		8 (47.1%)	8 (47.1%)	8 (47.1%)	1.0	1.0	1.0
	Interobserver agreement *Kappa*	1	0.88	0.88			
	Preferred sequence (R1/R2)	15/16	0/0	2/1			
**Vascular enhancement**		-	-	17 (100%)			
	Interobserver agreement *Kappa*	-	-	1			

Numbers in parentheses are percentages; “-“, not evaluated; *P*1, *P* values for comparison between PD and iMSDE-T1; *P*2, *P* values for comparison between PD and iMSDE-CE-T1; *P*, *P* values for comparison between iMSDE-T1 and iMSDE-CE-T1; R1, Reader 1; R2, Reader 2.

**Fig 1:**
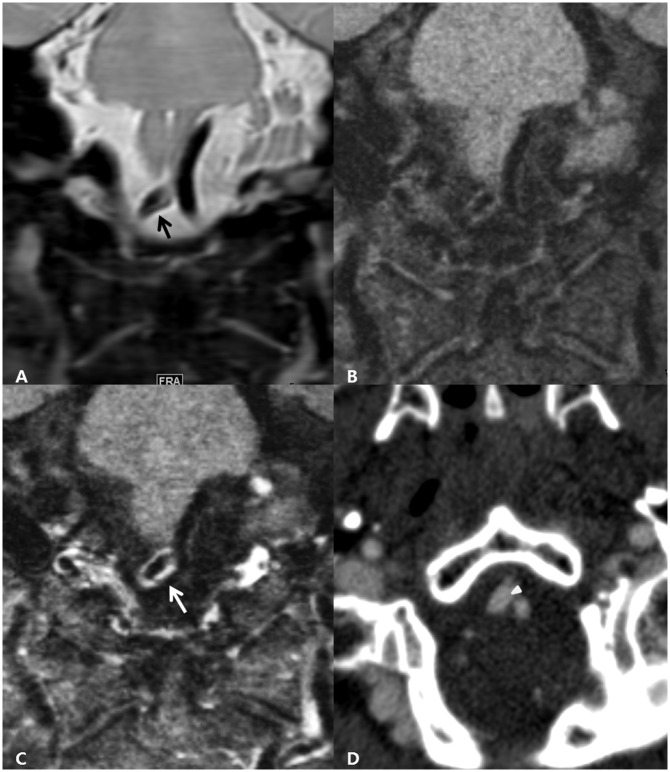
A 42-year-old man presenting with a headache. The coronal image of 3D-PD (A) clearly demonstrates the intimal flap (black arrow) which is not identified on the pre- (B) and post-contrast (C) 3D-iMSDE-T1 images. Abnormal vascular enhancement (white arrow) is revealed on the coronal post-contrast 3D-iMSDE-T1 image. The CTA source image (D) shows the intimal flap (white arrowhead).

**Fig 2:**
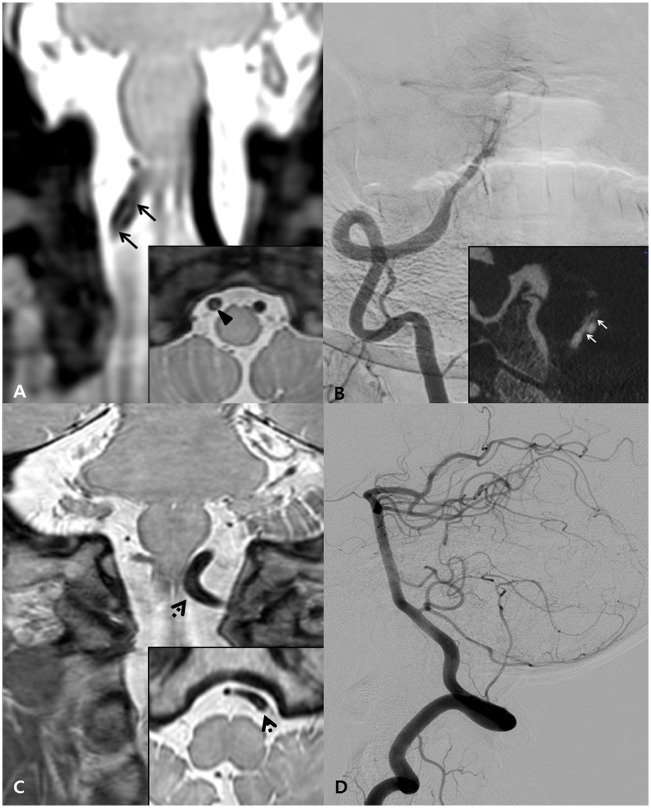
Representative images of a true intimal flap and flow artifact. A-B. Intimal flap. The coronal 3D-PD image (A) shows the intimal flap (black arrows) as a linear structure crossing the arterial lumen with clear continuity to the arterial wall (inbox, arrow head). Anteroposterior view of right VA angiography (B) shows irregular luminal narrowing and VasoCT (inbox) shows the intimal flap on a coronal plane image (white arrows). C-D. Flow artifact. The coronal 3D-PD image (C) shows a curvilinear structure (dotted arrow) which gradually fades away towards the endpoint without continuity with the arterial wall. Lateral view of left VA angiography (D) shows normal findings.

According to the diagnostic criteria, only eight (47.1%) patients were diagnosed with definite dissection on the pre and post-contrast 3D-iMSDE-T1 sequences. However, an intimal flap, one of the major criteria of intracranial dissection, was detected in 15 (88.2%) patients on the 3D-PD sequence (Fig 1). Therefore, significantly more patients could be diagnosed with definite dissection using the intimal flap imaging findings on 3D-PD, without additional imaging studies or follow-up (*P = 0*.*039*, Table 4). Among sixteen patients, who were assessed to evaluate the frequency of the intimal flap in the contralateral non-dissecting side, two (12.5%) showed flow artifacts. However, there were no false-positive findings for intimal flaps (Fig 2).

**Table 4:** Diagnosis of dissection using 3D-HR-MRI with or without 3D-PD.

	Definite dissection	Probable dissection	*P* value
iMSDE-T1 only	8 (47.1%)	9 (52.9%)	0.039
iMSDE-T1 + 3D-PD	15 (88.2%)	2 (11.8%)	

## Discussion

Our results showed that 3D-HR-MRI can demonstrate signs of intracranial arterial dissection. Furthermore, when the 3D-PD sequence was added, intimal flaps that would have been overlooked on 3D-iMSDE-T1 could be detected and definite dissection could be diagnosed without further invasive studies or follow-up.

Extracranial arterial dissection is known to be more common than intracranial arterial dissection [3, 4]. However, in the East Asian population, cervicocephalic arterial dissection occurs at least 2–3 times more often in intracranial arteries than extracranial arteries [21]. Additionally, in comparison with extracranial arterial dissection, intracranial arterial dissection is more likely to cause brain ischemia as well as subarachnoid hemorrhage and has poorer clinical prognosis [22–24]. Therefore, the diagnosis of intracranial arterial dissection is important, especially in certain ethnic populations.

Previous studies have reported that 3D-HR-MRI is helpful in the diagnosis of arterial dissection. Although some studies emphasize the detection of hyper-intense lesions on T1-weighted sequences which suggest IMH as evidence of arterial dissection [12, 25], the imaging findings of IMH might be influenced by chronological changes [26, 27]. As IMH might be iso-intense to the vessel wall in the acute or chronic stage [26, 27], it can be missed on T1-weighted images in these stages. Furthermore, intracranial atherosclerotic disease and intraplaque hemorrhage can show hyper-signal intensity on T1-weighted sequences [28] which may cause radiologists to confuse this with hyper-intense IMH on T1-weighted sequences.

Additionally, the intimal flap might be the most valuable imaging finding in dissection diagnosis because it is the only imaging finding used to diagnose definite dissection on noninvasive imaging modalities. In our study, intimal flaps were detected more often on the 3D-PD sequence than the pre- and post-contrast 3D-iMSDE-T1 sequences. The PD-weighted sequence has a high SNR, which can help detect these intimal flaps. On the other hand, the iMSDE-T1 sequence has a low SNR and its image quality is particularly vulnerable to patients’ motion which frequently occurs in patients with dissection due to severe headache, cerebral infarction, or subarachnoid hemorrhage [29]. Although the detection rate of intimal flaps on post-contrast iMSDE-T1 slightly improved due to contrast enhancement and increased signal intensity, this improvement did not show statistical significance in our study.

Several previous studies using the pre- or post-contrast T1-weighted sequences showed contradictory results on the detection rate of intimal flaps with a wide range of 6–91% [9, 13, 14]. Han et al. [9] reported relatively high detection rates of 48.6% and 91.4% on the pre- and post-contrast T1 sequences, respectively. Perhaps the subtle differences in the definition of the intimal flap could be the reason for this difference. In the report by Han et al., the definition of the intimal flap encompassed not only the wall between the true and false lumen but also the wall between IMH and the lumen. However, in our study, the intimal flap was more strictly defined as a linear structure crossing the arterial lumen located between the patent’s true and false lumen to exclude vessel walls overlaid with intraplaque hemorrhage within an atherosclerotic lesion. Natori et al. [14] revealed a very low detection rate of 6.3% for the intimal flap on the pre-contrast 3D T1-weighted sequence. The low detection rate might be attributed to a low SNR, which could be induced by using a 1.5T scanner and small voxel size (0.5mm isovoxel). In contrast, Sakurai et al. [13] revealed a relatively high detection rate (65%) for the intimal flap despite using a 1.5T scanner, which might be attributed to an improved SNR from larger voxel size (0.7x0.7x0.9mm).

Unexpectedly, in one patient, the intimal flap was detected on 3D-iMSDE-T1, but not on 3D-PD. In this case, we could not clearly distinguish the intimal flap from the vascular wall, so we regarded it as a flow artifact on 3D-PD. There can be several possible explanations for this. First, because the acquired voxel size of 3D-PD was larger than 3D-iMSDE-T1 and the intimal flap was detected on 3D-PD with 0.3 mm isovoxel resolution which was obtained in some patients including this patient, the partial volume effect might have contributed to this false-negative finding. Further study to assess the role of spatial resolution as well as the SNR in diagnosing intracranial arterial dissection is warranted. Second, the patient might have slightly moved during 3D-PD scanning, which could degrade the image quality. In this patient, the intimal flap was not detected on post-contrast 3D-iMSDE-T1 as well, even though identical imaging parameters were applied and an improved SNR of the intimal flap was expected. We speculated that this might be due to subtle motion as we obtained post-contrast 3D-iMSDE-T1 at the end of the protocol and more motion could have occurred, affecting the image quality of small structures such as intimal flaps.

In the contralateral non-dissection side, we did not find false-positive findings for the intimal flap on 3D-PD. However, flow artifacts which can mimic intimal flaps were found in two patients. Therefore, knowledge about the definition and corresponding imaging findings of intimal flaps and flow artifacts on 3D-PD is required to prevent overdiagnosis of dissection.

The relatively small number of subjects and retrospective study design are limitations of our study. In addition, not all patients underwent conventional angiography which is the current gold standard modality for diagnosis of arterial dissection. However, we diagnosed definite dissection according to published diagnostic criteria as described in previous studies [15]. Finally, we did not compare image findings between patients with intracranial dissection and normal controls or between patients with intracranial dissection and patients with other vascular diseases (e.g., atherosclerosis). Therefore, diagnostic performance of 3D-HR-MRI for intracranial arterial dissection could not be fully evaluated. Future research with a large number of patients and a proper control group is needed to validate our results.

In conclusion, 3D-HR-MRI may be useful for diagnosing intracranial arterial dissection particularly when 3D-PD is added, allowing detection of intimal flaps that could be overlooked with 3D-iMSDE-T1 alone.
